# Tropism of *Puumala orthohantavirus* and Endoparasite Coinfection in the Bank Vole Reservoir

**DOI:** 10.3390/v15030612

**Published:** 2023-02-23

**Authors:** Elfi K. Schlohsarczyk, Stephan Drewes, Paweł Koteja, Susanne Röhrs, Rainer G. Ulrich, Jens P. Teifke, Christiane Herden

**Affiliations:** 1 Institute of Veterinary Pathology, FB10—Veterinary Medicine, Justus-Liebig-University Giessen, 35392 Giessen, Germany; 2 Friedrich-Loeffler-Institut, 17493 Greifswald-Insel Riems, Germany; 3 Institute of Environmental Sciences, Faculty of Biology, Jagiellonian University, 30-387 Kraków, Poland

**Keywords:** *Puumala orthohantavirus*, hantavirus infection, zoonoses, rodents, bank vole, immunohistochemistry, in situ hybridization, endoparasite coinfection

## Abstract

In Europe, most cases of human hantavirus disease are caused by *Puumala orthohantavirus* (PUUV) transmitted by bank voles (*Clethrionomys glareolus,* syn. *Myodes glareolus*), in which PUUV causes inconspicuous infection. Little is known about tropism and endoparasite coinfections in PUUV-infected reservoir and spillover-infected rodents. Here, we characterized PUUV tropism, pathological changes and endoparasite coinfections. The voles and some non-reservoir rodents were examined histologically, immunohistochemically, by in situ hybridization, indirect IgG enzyme-linked immunosorbent assay and reverse transcription-polymerase chain reaction. PUUV RNA and anti-PUUV antibodies were detected simultaneously in a large proportion of the bank voles, indicating persistent infection. Although PUUV RNA was not detected in non-reservoir rodents, the detection of PUUV-reactive antibodies suggests virus contact. No specific gross and histological findings were detected in the infected bank voles. A broad organ tropism of PUUV was observed: kidney and stomach were most frequently infected. Remarkably, PUUV was detected in cells lacking the typical secretory capacity, which may contribute to the maintenance of virus persistence. PUUV-infected wild bank voles were found to be frequently coinfected with *Hepatozoon* spp. and *Sarcocystis* (*Frenkelia*) spp., possibly causing immune modulation that may influence susceptibility to PUUV infection or vice versa. The results are a prerequisite for a deeper understanding of virus–host interactions in natural hantavirus reservoirs.

## 1. Introduction

Various rodents play an important role as natural hosts for hantaviruses, but in recent years, hantaviruses have also been found in insectivores and bats [1]. Typically, each hantavirus species is associated with only one rodent species [2]. In Central, Western and Northern Europe, most human cases of hantavirus disease are caused by Puumala orthohantavirus (PUUV) transmitted by the bank vole (*Clethrionomys glareolus* syn. *Myodes glareolus*) [3]. Spillover infections are rarely observed in yellow-necked mice (*Apodemus flavicollis*) and field voles (*Microtus agrestis*) [4,5]. Infected bank voles shed the virus via saliva, urine and feces [6], and infection usually occurs through the inhalation of virus-contaminated dust or through bites [7]. PUUV causes mild to moderate hemorrhagic fever with renal syndrome in humans, also called nephropathia epidemica [8]. Hantaviruses are thought to have adapted to their reservoir host during coevolution [9,10,11]. Therefore, the infection in the reservoir is clinically silent [12]. Evasion, suppression or modification of the host immune response may be a reason for virus persistence in the reservoir [13]. A deficiency in immuneregulatory processes can lead to hantavirus disease in humans [14]. As with all orthohantaviruses, PUUV is an enveloped virus with a segmented RNA genome of negative polarity [15]. The small (S) segment of 1826–1830 nucleotides encodes the nucleocapsid (N) protein, and in an overlapping open reading frame, a non-structural (NSs) protein [16,17,18]. The medium (M) segment of 3682 nucleotides codes for a glycoprotein precursor (GPC) that is co-translationally cleaved into an N-terminal (Gn) and a C-terminal (Gc) part [19]. The large (L) segment of 6530–6562 nucleotides encodes the L protein with the RNA-dependent RNA polymerase [20]. The S, M and L segments are frequently used for diagnostic purposes and classification of PUUV lineages [4,21,22,23,24]. The serological detection of PUUV infections in bank voles can be performed by an immunoglobulin G (IgG) enzyme-linked immunosorbent assay (ELISA) using recombinant N protein [21]. The various PUUV strains have been classified into several clades [25,26,27]. These clades are associated with certain evolutionary lineages of the bank vole [28,29,30]. In Germany, the Central European clade of PUUV has been identified in association with the Western evolutionary lineage of the bank vole [28]. The district Osnabrück (OS) in the north-western part of Germany has long been known to be an endemic area for PUUV [25,28,31]. Depending on the geographical region, the Latvian and Russian PUUV clades were identified in Poland, which are connected with the Eastern and Carpathian evolutionary lineages of the bank vole [32,33].

To date, little is known about the cell tropism of PUUV in its natural host, the bank vole, the modes of transmission and the possible role of endoparasitic coinfections in the course of PUUV infection. Coinfections may increase the likelihood of PUUV infection with subsequent viral replication and shedding through modulation of the immune response [34,35]. In addition, the reasons for the lack of obvious clinical signs in the infected reservoir host, unlike in humans, have been largely unclear. Therefore, the objectives of this study were the following: (1) to characterize the tropism of PUUV of the Central European and Russian clades in two different groups of naturally infected voles from the Western and Carpathian evolutionary lineages to improve the understanding of the course and transmission routes in these animals; (2) to identify possible PUUV-associated pathological changes as well as endoparasite coinfections in both vole cohorts; (3) to screen non-reservoir rodents for spillover infections.

## 2. Materials and Methods

### 2.1. Samples and Study Design

In this study, 192 bank voles, 95 males and 93 females (four animals could not be assigned to a sex); 221 yellow-necked mice, 118 males and 102 females (one animal could not be assigned to a sex); and 39 wood mice (*A. sylvaticus*), 23 males and 16 females, trapped between 2015 and 2017 in the district of OS in Lower Saxony, north-west Germany, were investigated. Thirteen additional female bank voles originated from a breeding facility (BF) of bank voles. The animals were not kept under strict hygienic conditions and were not given any antiparasitic drugs. PUUV was accidentally introduced into the bank vole colony by infected bank voles taken from a wildlife population in south-eastern Poland (see below). The origin and maintenance conditions of the long-term breeding colony, as well as the work associated with the subsequent transfer of animals captured in the wild to the same facility, have been described previously [36,37]. As explained in Sadowska et al. 2015 [36], the infection of the colony with PUUV was discovered much later, so no further details on the route of transmission can be given. However, since the wild-trapped animals were never kept in the same cages or even the same rooms as the animals from the permanent colony, direct animal-to-animal transmission was not possible, so the only feasible route was indirect via dust.

Both groups potentially differed in age, nutritional and immune status, genetic variability and metagenome, and were therefore considered as different naturally infected groups. A non-PUUV-infected male bank vole from the breeding facility of the Friedrich-Loeffler-Institut served as negative control. All animals were dissected according to a standard protocol; native samples of lung, other tissues (Table S1) and thoracic fluid samples were obtained and stored at −20 °C until use. In parallel, tissue samples were prepared for subsequent histopathological examination and virus detection (see below). The weight of the bank voles was used as a proxy for the age; animals of <15 g were considered as juvenile [38].

The rodents were initially screened by indirect PUUV IgG ELISA and reverse transcription-polymerase chain reaction (RT-PCR) and classified into PUUV RNA-positive/anti-PUUV-IgG-positive, PUUV-RNA-negative/anti-PUUV-IgG-positive, PUUV RNA-positive/anti-PUUV-IgG-negative, and PUUV RNA-negative/anti-PUUV-IgG-negative. Thereafter, groups of these animals were evaluated by histology, immunohistochemistry (IHC) and in situ hybridization (ISH) (Figure S1). The total number of animals or number of animals investigated for a certain organ could vary according to the post-mortem decomposition of the respective organs and due to a change in the number of samples collected during the course of the study (Table S1).

### 2.2. Indirect IgG ELISA and S Segment-Specific RT-PCR for Detection of Anti-PUUV-IgG Antibodies and PUUV RNA

Chest cavity fluid samples from bank voles, yellow-necked mice and wood mice were examined by IgG ELISA using recombinant purified N protein of the PUUV strain Bavaria, as described elsewhere [5]. The N protein-specific monoclonal antibody 5E11 served as positive control [39,40]. Chest cavity fluid samples of PUUV RT-PCR-negative and IgG ELISA-negative bank voles and yellow-necked mice/wood mice were used as negative controls for the serology of bank voles and *Apodemus* mice, respectively. Differentiation between positive, negative and equivocal results was performed according to a previously described decision tree [41]. For PUUV RNA detection, nucleic acid was extracted from homogenized lung tissue [42] using QIAzol Lysis Reagent (QIAGEN, Hilden, Germany) followed by a “one step” S segment-specific RT-PCR. The primers 342F (5′-TATGGTAATGTCCTTGATGT-3′) and 1102R (5′-GCCATDATDGTRTTYCTCAT-3′) were used to amplify the main region of the N protein-coding sequence [4]. The cell culture supernatant of cells infected with the PUUV strain Sotkamo served as positive control. The RT-PCR assay and sequencing were performed as described previously [5] (and see below).

### 2.3. Phylogenetic Analyses of PUUV and Cytochrome b Sequences

The phylogenetic analyses of PUUV and cytochrome *b* sequences were performed as described previously [5]. The appropriate substitution model for calculating a phylogenetic tree was determined using jModel Test2 on XSEDE (2.1.6) [43] of the platform Cyberinfrastructure for Phylogenetic Research (CIPRES) Science Gateway V.3.3, (https://www.phylo.org/, accessed on 10 January 2023) [44]. Subsequently, a Bayesian tree was calculated with MrBayes on XSEDE (3.2.7a) [45], followed by further processing using FigTree (1.4.2.). Then, a maximum likelihood tree was calculated using FastTreeMP on XSEDE (2.1.10) [46]. Finally, the data of the Bayesian and maximum likelihood trees were merged to a consensus phylogenetic tree and the PUUV- and cytochrome *b* sequences were assigned to their respective clades.

### 2.4. Hematoxylin and Eosin Staining (H&E) and Immunohistochemistry (IHC) for Detection of Histopathological Changes and PUUV N-Protein

Tissues from 38 bank voles, 4 yellow-necked mice, 2 wood mice from OS, 13 bank voles from BF and the negative control animal were fixed in 10% neutral buffered formalin (NBF) for 24 h and then processed, embedded in paraffin wax and sectioned at 3 µm. The tissue sections were deparaffinized and rehydrated with graded alcohols. Tissues were then H&E and immunostained. For IHC, endogenous peroxidase was inhibited by incubation in a 0.5% hydrogen peroxide (H_2_O_2_) solution (Merck KGaA, Darmstadt, Germany) for 30 min at room temperature. This was followed by pretreatment with 0.05% proteinase K solution (Carl Roth GmbH + Co. KG, Karlsruhe, Germany) at 37 °C for 6 min. Subsequently, the sections were exposed to a 1:1500 dilution of a hyperimmune pig antiserum raised against recombinant PUUV N protein and normal pig serum with the same dilution as negative control for 24 h at 4 °C. The pig sera originated from an immunization study at the Friedrich-Loeffler-Institut [47]. The next steps consisted of blocking endogenous biotin using the Avidin/Biotin Blocking Kit (Vector Laboratories, Burlingame, CA, USA) according to the manufacturer’s instructions, 30 min incubation at room temperature with a 1:500 dilution of a biotinylated goat anti-pig secondary antibody (Jackson ImmunoResearch Laboratories, Inc., West Grove, PA, USA), 30 min incubation with the Avidin-Biotin-Complex using the Vectastain Elite ABC Kit Standard (Vector Laboratories, Burlingame, CA, USA) according to the manufacturer’s instructions, and 2 min incubation at room temperature with 0.05% 3,3′-diaminobenzidin-tetrahydrochlorid-dihydrate (DAB; Merck KGaA, Darmstadt, Germany) for visualization of the reaction. After counterstaining with a 1:10 dilution of Papanicolaou (Merck KGaA, Darmstadt, Germany) for 15 s at room temperature, sections were dehydrated and placed on a coverslip with Tissue Tek Film (Sakura Finetek GmbH, Staufen, Germany).

### 2.5. In Situ Hybridization (ISH) for Detection of Positive-Strand RNA

To prepare a probe for detecting PUUV positive-strand RNA, nucleic acid was isolated [5] from the lung tissue of 10 BF bank voles. Then, an RT-PCR assay using the primers 342F and 1102R (see Section 2.2) was performed. The sequencing reactions were carried out with the same primers using the Big Dye Terminator v1.1. Cycle Sequencing Kit (Thermo Fisher Scientific, Waltham, MA, USA) according to the manufacturer’s instructions. Subsequently, the products were purified using the NucleoSEQ Kit (Macherey-Nagel GmbH & Co. KG, Düren, Germany) according to the manufacturer’s instructions and sequenced using the 3130 Genetic Analyzer (Applied Biosystems, Thermo Fisher Scientific, Waltham, MA, USA). For every bank vole, a consensus sequence was generated using BioEdit (BioEdit Sequence Alignment Editor, version 7.2.5) [48,49]. Finally, a consensus sequence of all individual PUUV consensus sequences was created and sent to the manufacturer (Advanced Cell Diagnostics, Newark, NJ, USA) for generating 20 double-Z-probe pairs (RNAscope Probe-V-PUUV-N) for detecting the PUUV positive-strand RNA. As negative control, double-Z-probe pairs (RNAscope Negative Control Probe-DapB; Advanced Cell Diagnostics, Newark, NJ, USA) detecting the Dap B gene of the soil bacterium *Bacillus subtilis* subspecies *subtilis* strain *subtilis Marburg Yale* were used. PUUV-infected Vero E6 cells were used as positive control. As with the tissue samples, the cells were fixed in NBF and embedded in paraffin wax.

For each RNA detection probe, a section was cut from the respective formalin-fixed paraffin-embedded tissues. The ISH was performed with four bank voles from BF and the negative control vole according to the manufacturer’s instructions [50].

## 3. Results

### 3.1. Serological Analyses for Anti-PUUV-IgG, Demonstration of PUUV RNA by S RT-PCR and Phylogenetic Analyses of PUUV and Cytochrome b Sequences

Of the investigated 188 bank voles, 36 (18/92, 19.6%, males and 18/93, 19.4% females) were found to be viral RNA- and antibody-positive. This indicates a similar proportion of persistently infected males and females. Three (2/92 males and 1/93 females) were viral RNA-positive and antibody-negative, indicating an acute infection. In addition, 21 (7/92, 7.6% males and 14/93, 15.1%, females) tested antibody-positive but RNA-negative, and one male out of 92 as antibody-equivocal and RNA-negative. Of these 21 animals, two (one male, one female) were juveniles (weight < 15 g) and therefore might have carried maternal antibodies, whereas the remaining 19 animals (6 males, 13 females) might indicate viral clearance. Finally, 128 (64/92 males and 60/93 females) voles were RNA- and antibody-negative. Among the 13 voles from BF, all female, 12 tested positive for viral RNA and anti-PUUV antibodies (Table 1). One BF vole was antibody-positive but tested negative for PUUV RNA by RT-PCR. Eighteen of 220 yellow-necked mice and 7 of 39 wood mice were anti-PUUV IgG reactive, but in none of the mice PUUV RNA could be amplified. All RNA- and antibody-positive voles from OS were assigned to the Western evolutionary lineage and the animals from BF to the Carpathian lineage (Figure S2). The PUUV S segment sequences from OS were classified as the Central European clade and those from BF as the Russian clade (Figure S3). The PUUV sequences from the BF showed a 99.7% similarity over a length of 465 nucleotides to sequences from southern Poland, suggesting an incursion of the PUUV infection by wild-trapped bank voles.

### 3.2. Detection of PUUV N-Protein by IHC, Viral Positive-Strand RNA by ISH, Gross and Histologic Findings and Endoparasite Coinfections

No specific gross lesions occurred in any animal of the wild or laboratory animal group, independently of the infection status.

Based on the results of the RT-PCR and serological screening, bank voles from OS and BF were selected for examination by IHC (Table 1), ISH (Table 2) and H&E staining (Table 3, Table 4, Table 5, Table 6 and Table 7).

Generally, the N protein was detected by IHC in 22/32 (12 males, 10 females) and 9/12 of the RT-PCR-positive bank voles from OS and BF, respectively (Table 1). The organ-specific and cellular expression patterns did not differ in the two different animal cohorts. No N protein was detected in the antibody-positive but RNA-negative BF vole.

**Figure 1:**
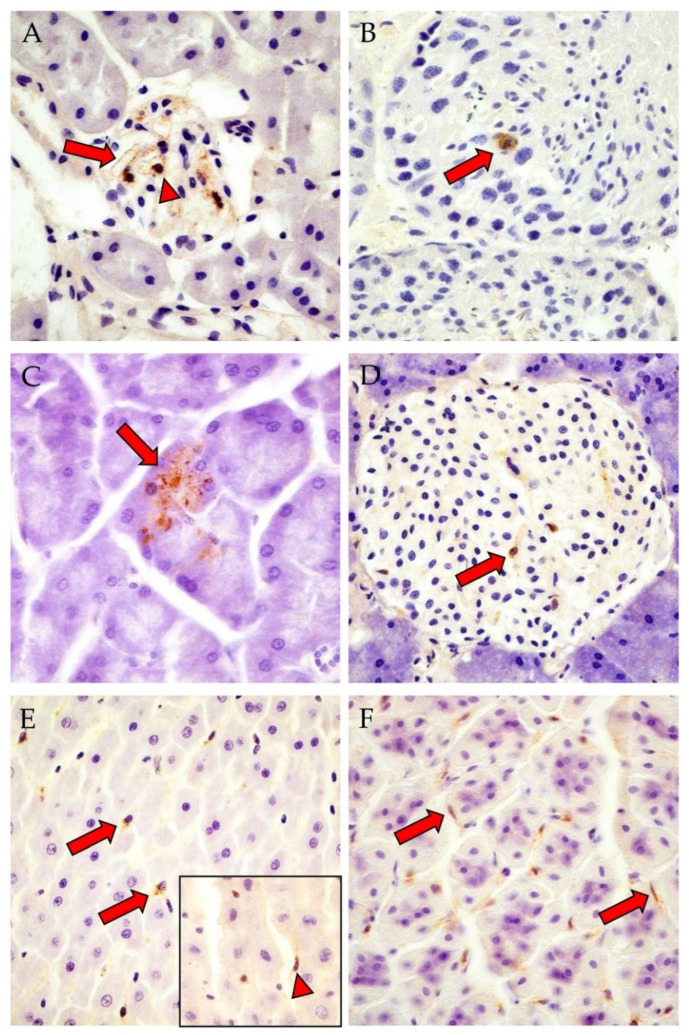
Detection of PUUV nucleocapsid (N) protein in different tissues of OS (**A**,**B**,**D**,**E**, inset) and BF (**C**,**F**) bank voles by IHC. (**A**): male, RNA-positive, antibody-negative, kidney: positive endothelial cells (red arrow) and glomerulum cells (red arrowhead); (**B**): male, RNA-positive, antibody-positive, testis: sperm precursor cell with viral antigen (red arrow); (**C**): female, RNA-positive, antibody-positive, exocrine pancreas: positive acini (red arrow); (**D**): male, RNA-positive, antibody-positive, endocrine pancreas: positive islet cells of Langerhans (red arrow); (**E**): male, RNA-positive, antibody-negative, liver: positive Kupffer cells (red arrows); inset: female, RNA-positive, antibody-negative, N protein in cells with elongated spindle-shaped nuclei at sinusoid periphery (red arrowhead); (**F**): female, RNA-positive, antibody-positive, pars glandularis of the stomach: viral antigen in interstitial cells with lancet to spindle-shaped nuclei (red arrows). Total magnification: 400×. Corresponding positive signals in OS or BF voles are shown in Figure S5. IHC images of kidney, pancreas, liver and pars glandularis of the stomach of the negative control vole are shown in Figure S4.

The viral antigen was most abundant in the kidney (OS: 14/31, BF: 4/12) and in the pars glandularis of the stomach (OS: 5/13, BF: 8/11) (Table 1, Figure 1A,F). Only in the bank voles from OS the N protein was found in the glandula mandibularis, tongue, liver and heart, whereas the glandula parotidea was exclusively tested positive in the female animals of BF (Table 1). Notably, the N protein was detected in the testis of a vole from OS. Since the BF voles were exclusively female, no conclusion can be drawn with regard to the testicles of male animals of the Carpathian lineage as a target organ for PUUV.

Two antibody-positive and RNA-negative bank voles from OS tested negative in the IHC (Figure S4).

**Figure 2:**
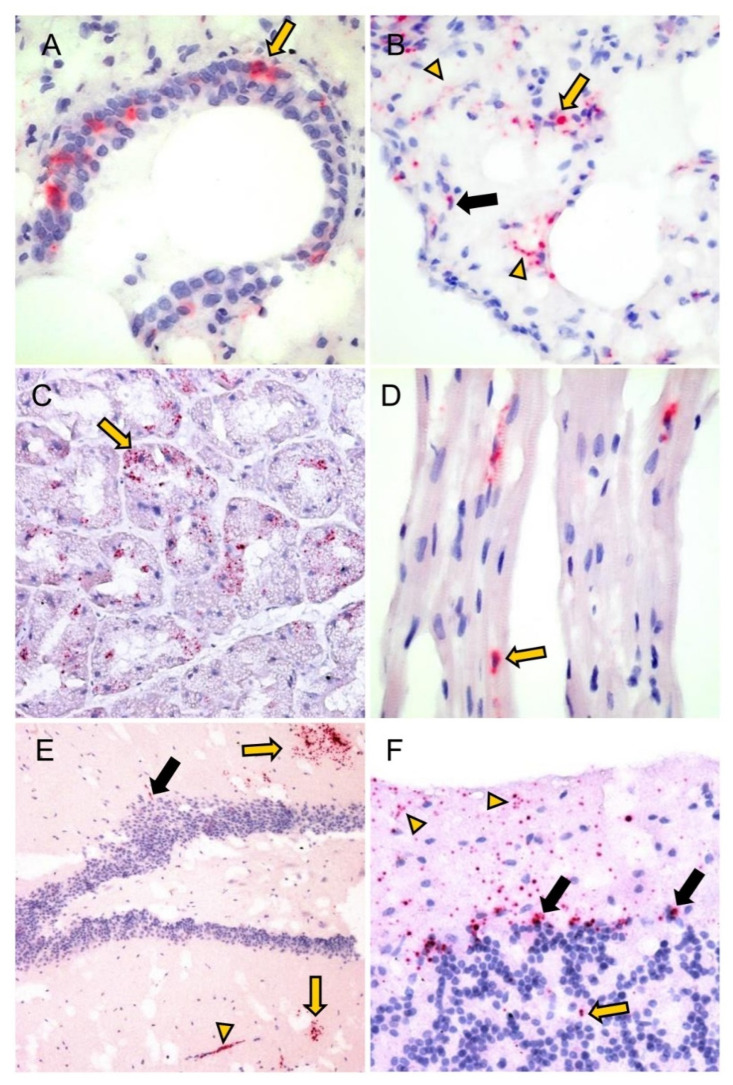
Detection of positive-strand PUUV RNA in various tissues of BF bank voles by RNAscope. (**A**–**F**): female, RNA-positive, antibody-positive; (**A**) lung: positive-strand RNA in bronchiolar epithelial cells (yellow arrow); (**B**) lung: positive signals in interstitial cells with spindle-shaped (black arrow) and round-oval nuclei (yellow arrow) as well as fine, strand-like signals in the interstitium and in the alveolar walls (yellow arrowheads); (**C**) Glandula submandibularis: positive acini (yellow arrow); (**D**) heart: positive signals in cardiomyocytes (yellow arrow); (**E**) cerebrum: positive glia cells (yellow arrows), endothelial cells (yellow arrowhead) and neurons (black arrow); (**F**) cerebellum: positive-strand RNA in glia cells (yellow arrowheads), Purkinje cells (black arrows) and granule cells (yellow arrow). Total magnification: (**A**,**B**,**D**,**F**): 400×, (**C**):200×, (**E**):100×. Results of the RNAscope analysis of lung, glandula submandibularis, heart, cerebrum and cerebellum of the negative control vole are shown in Figure S6.

Using the ISH method, the PUUV positive-strand RNA was detected in the kidney (not shown) and lung (Figure 2A,B) of 4/4 BF bank voles (Table 2). The N protein and the positive-strand viral RNA were colocalized in serial sections of both groups of bank voles from OS and BF.

Generally, the expression patterns of N protein and positive-strand RNA were similar in both animal cohorts; they were detected predominantly in mesenchymal cells, less frequently in epithelial and neuroectodermal cells and rarely in endocrine cells (Table 1 and Table 2; Figure 1 and Figure 2). Pulmonary or gastric macrophages, renal podocytes or mesangial cells (interstitial cells with round to oval nuclei), endothelial cells or fibrocytes, gastric and intestinal myocytes (interstitial cells with lancet to spindle-shaped nuclei), hepatic Ito cells and myoepithelium of the mandibular gland were positive for N protein and positive-strand RNA (Figure 1 and Figure 2). Furthermore, the N protein was detected in a sperm precursor cell of a single OS bank vole (Figure 1B), whereby positive-strand RNA, but no N protein was detected in myocytes (tongue, heart), adrenal endocrine cells as well as in interstitial cells in the kidney, adrenal gland, intestine (cecum, colon ascendens and colon descendens) and brown adipose tissue (Table 2; Figure 2). Overall, N protein and positive-strand RNA were only detected in a few cells within an organ.

**Table 2:** Detection of Puumala orthohantavirus (PUUV) RNA by RT-PCR, anti-PUUV IgG antibodies by ELISA and RNAscope in situ hybridization (ISH)-based detection of positive-strand RNA in bank voles from the breeding facility (BF).

		**No. of Positive/Total Number of Investigated Voles**	
		**RT-PCR Pos** **ELISA Pos** **(Persistent Infection)**	**RT-PCR Neg** **ELISA Pos**
		**BF**	**BF**
		12/13	1/13
		**Detection of Viral Positive-Strand RNA by ISH**	
Total		3/4 (75%)	1/1 (100%)
Cerebrum (neurons, glia cells, endothelial cells, plexus choroideus [cell with lancet-shaped nucleus in the pia mater])		1/3 (33%)	0/1 (0%)
Cerebellum (Stratum moleculare, -ganglionare and -granulosum)		1/3 (33%)	0/1 (0%)
Lung		3/3 (100%)	1/1 (100%)
(a) Bronchiolar epithelial cells		1/3 (33%)	0/1 (0%)
(b) Pneumocytes type I and II (c) Endothelial cells		3/3 (100%)	1/1 (100%)
		1/3 (33%)	0/1 (0%)
(d) IC with spindle-shaped and round-oval nuclei		3/3 (100%)	1/1 (100%)
Glandula submandibularis (acini)		1/2 (50%)	n.i.
Glandula mandibularis (acini)		1/1 (100%)	n.i.
Glandula parotidea (acini)		1/1 (100%)	n.i.
Tongue (myocytes, IC with lancet to spindle-shaped nuclei)		1/1 (100%)	n.i.
Liver (Kupffer cells)		2/3 (66%)	0/1
Pancreas (a) Acini (b) Islet cells of Langerhans (c) IC with spindle-shaped and round-oval nuclei		3/3 (100%)	n.i.
		3/3 (100%)	n.a.
		1/3 (33%)	n.a.
		2/3 (66%)	n.a.
**Stomach/Pars non-glandularis (epithelial)**		2/2 (100%)	0/1 (0%)
**Stomach/Pars glandularis** (IC with lancet to spindle-shaped and round-oval nuclei)		2/2 (100%)	0/1 (0%)
**Duodenum**	IC with spindle-shaped nuclei	3/3 (100%)	0/1 (0%)
**Caecum**		1/1 (100%)	n.i.
**Colon ascendens**		1/1 (100%)	n.i.
**Colon descendens**		2/3 (66%)	0/1
**Kidney** (a) Glomerulum cells * (b) IC with spindle-shaped nuclei (cortex)		3/3 (100%)	1/1 (100%)
		3/3 (100%)	1/1 (100%)
		2/3 (66%)	0/1 (0%)
**Heart** (kardiomyocytes)		2/3 (66%)	1/1 (100%)
**Adrenal gland** (endocrine cells, IC with lancet to plump, spindle-shaped nuclei (cortex and medulla))		1/1 (100%)	n.i.
**Brown adipose tissue** (interstitium) **		1/1 (100%)	n.i.

No PUUV positive-strand RNA was detected in the negative control bank vole. IC, interstitial cells; *, glomerulum cells include endothelial cells, mesangial cells and podocytes; **, affected cell type not identifiable; n.a., not applicable; n.i., not investigated.

In line with the results of the RT-PCR-investigation, the PUUV antigen was not found in the yellow-necked and wood mice and the negative control bank vole (Figures S6 and S7).

No specific histologic lesions occurred in any of the animals investigated.

Several bank voles from OS, in which the PUUV RNA and anti-PUUV antibodies were detected or which were only viral RNA-positive, showed infiltrates of mononuclear cells in the urinary tract, salivary gland, heart and brown adipose tissue. The latter finding also occurred in a PUUV RNA and anti-PUUV antibody-negative bank vole (Table 3). No findings occurred in animals that were exclusively serologically positive.

**Table 3:** Histologic findings in male and female bank voles from OS.

	RT-PCR Pos ELISA Pos (Persistent Infection)	RT-PCR Pos ELISA Neg (Acute Infection)	RT-PCR Neg ELISA Neg
Total	14/15 M, 1/15 F	2/3 M, 1/3 F	2/2 F
Kidney (a) Mild chronic non-suppurative interstitial nephritis (b) Few interstitial mononuclear cells (c) Few plasma cells in renal pelvis (d) Few plasma cells in perirenal adipose tissue			
	2/29 (7%)	0/3 (0%)	0/2 (0%)
	2/29 (7%)	0/3 (0%)	0/2 (0%)
	1/29 (3%)	0/3 (0%)	0/2 (0%)
	0/29 (0%)	1/3 (33%)	0/2 (0%)
Urinary bladder (a) Few interstitial plasma cells			
	1/17 (6%)	0/1 (0%)	0/2 (0%)
Glandula mandibularis (a) Few interstitial lymphocytes (b) Few interstitial lymphocytes and plasma cells			
	2/16 (13%)	1/1 (0%)	0/2 (0%)
	1/16 (6%)	0/1 (0%)	0/2 (0%)
Brown adipose tissue (a) Few monocytes (b) Few interstitial lymphocytes and plasma cells			
	0/16 (0%)	1/1 (100%)	0/2 (0%)
	0/16 (0%)	0/1 (0%)	1/2 (50%)
Heart (a) Few interstitial lymphocytes			
	1/29 (3%)	0/1 (0%)	0/2 (0%)

M, males; F, females.

**Figure 3:**
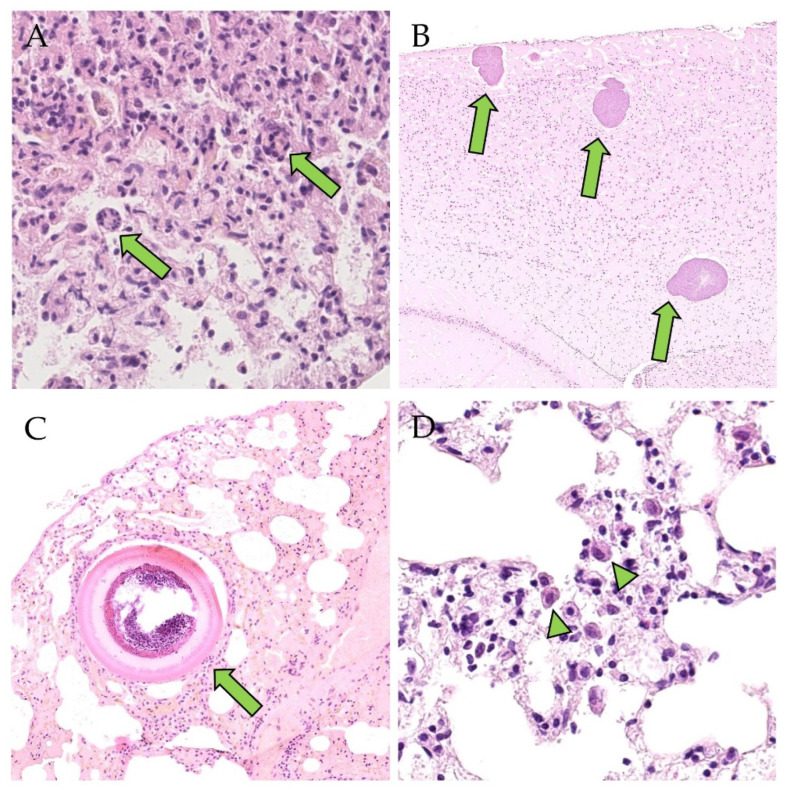
Endoparasites and fungal infection of OS bank voles (H&E). (**A**): male, RNA-negative, antibody-positive, lung: schizonts of *Hepatozoon* spp. (green arrows); (**B**): male, RNA-positive, antibody-positive, brain (cerebrum): cysts of *Sarcocystis* spp. without associated inflammation (green arrows); (**C**): female, RNA-positive, antibody-positive, lung: *Emmonsia crescens* (green arrow); (**D**): female, RNA-positive, antibody-positive, lung: desquamated alveolar macrophages (green arrowheads). Total magnification: (**A**,**D**): 400×, (**B**): 40×, (**C**): 100×.

Notably, every PUUV-RNA-positive bank vole from OS from which brain material was available (23/23) was co-infected with cysts (schizonts) of *Hepatozoon* spp. (8/23; Figure 3A) in the lung or *Sarcocystis* spp. (synonymous *Frenkelia* spp.) [51] (2/23) (Figure 3B) in the brain, or both protozoa were detected in the same animal (13/23) (Table 4). *Hepatozoon* spp. was also found in the lungs of 2/3 RNA-negative and antibody-positive OS voles. *Emmonsia crescens* (Figure 3C) was rarely detected in the lung. This fungus was accompanied by a mild granulomatous inflammation or did not react like *Sarcocystis* spp. in the brain. Almost all PUUV RNA and antibody-positive voles from OS (21/23) whose lungs were infected with *Hepatozoon* spp. also showed histologic lesions in the lung: desquamated alveolar macrophages (Figure 3D) and interstitial inflammatory cells (mononuclear cells, neutrophils, eosinophils) were almost always present, and rarely accompanied by syncytia or hyperplasia of bronchus-associated lymphoid tissue (BALT). The most severe lesion was interstitial pneumonia (Table 5). Neither positive-strand RNA nor N protein were detected within any described inflammatory changes.

The protozoa as well as *Emmonsia crescens*, diagnosed according to Gardiner et al. 1985 [52], were not detected in 2/2 OS voles negative for both PUUV RNA and anti-PUUV antibodies. Nevertheless, pulmonary interstitial mononuclear infiltrates and neutrophils were found in two PUUV RNA and antibody-negative bank voles, and additional desquamated alveolar macrophages were found in one of these animals.

**Table 4:** Detection of *Hepatozoon* spp. in the lung or *Sarcocystis* spp. in the brain or both endoparasites by hematoxylin eosin (H&E) staining in bank voles from OS compared with detection of PUUV RNA by RT-PCR and anti-PUUV antibodies by ELISA.

	*Hepatozoon* spp.	*Sarcocystis* spp.	*Hepatozoon* spp. + *Sarcocystis* spp.
RT-PCR pos, ELISA pos (persistent infection)	7/21 (33%) 5/7 M, 2/7 F	2/21 (10%) 1/2 M, 1/2 F	12/21 (57%) 6/12 M, 6/12 F
RT-PCR pos, ELISA neg (acute infection)	1/2 (50%) 1/1 M	0/2 (0%)	1/2 (50%) 1/1 M
RT-PCR neg, ELISA pos	2/3 (67%) 2/2 M	0/3 (0%)	0/3 (0%)
RT-PCR neg, ELISA neg	0/2 (0%)	0/2 (0%)	0/2 (0%)

M, males; F, females.

**Table 5:** Histologic findings in the lungs of bank voles from OS affected with *Hepatozoon* spp., *Emmonsia crescens* or both pathogens.

	RT-PCR Pos ELISA Pos (Persistent Infection)	RT-PCR Pos ELISA Neg (Acute Infection)	RT-PCR Neg ELISA Pos	Pathogen
**Total**	23/29 (79%) 12/23 M, 11/23 F	3/3 (100%) 2/3 M, 1/3 F	3/4 (75%) 3/3 M	*Hepatozoon* spp.
	1/29 (3%) 1/1 F	0/3 (0%)	0/4 (0%)	*E. crescens*
	4/29 (14%) 2/4 M, 2/4 F	0/3 (0%)	0/4 (0%)	*Hepatozoon* spp. and *E. crescens*
Desquamated alveolar macrophages	6/23 (26%)	1/3 (33)	1/4 (25%)	*Hepatozoon* spp.
Interstitial infiltrates: mononuclear cells *	1/23 (4%)	0/3 (0%)	1/4 (25%)	
Interstitial infiltrates: mononuclear cells, BALT-hyperplasia *	1/23 (4%)	0/3 (0%)	0/4 (0%)	
Interstitial infiltrates: mononuclear cells, neutrophils *	10/23 (44%)	0/3 (0%)	0/4 (0%)	
Interstitial infiltrates: mononuclear cells, neutrophils, syncytia *	0/23 (0%)	1/3 (33)	0/4 (0%)	
Interstitial infiltrates: mononuclear cells, neutrophils, eosinophils *	1/23 (4%)	1/3 (33)	0/4 (0%)	
Interstitial infiltrates: mononuclear cells, neutrophils, eosinophils, syncytia *	1/23 (4%)	0/3 (0%)	0/4(0%)	
Interstitial pneumonia, neutrophils, syncytia *	1/23 (4%)	0/3 (0%)	1/4 (25%)	
Desquamated alveolar macrophages	1/28 (4%)	0/3 (0%)	0/3 (0%)	*E. crescens*
Desquamated alveolar macrophages	1/4 (25%)	0/3 (0%)	0/3 (0%)	*Hepatozoon* spp. and *E. crescens*
Interstitial infiltrates: mononuclear cells, neutrophils	1/4 (25%)	0/3 (0%)	0/3 (0%)	
Interstitial infiltrates: mononuclear cells, neutrophils, eosinophils *	1/4 (25%)	0/3 (0%)	0/3 (0%)	

*, findings accompanied by desquamated alveolar macrophages; M, males; F, females.

Almost all persistently infected BF bank voles showed desquamated alveolar macrophages in the lung. Infiltrates of mononuclear cells were present in the lung, salivary gland and heart of a few persistently infected animals (Table 6). Neither positive-strand RNA nor N protein were detected within the inflammatory changes. None of the BF voles were affected with *Hepatozoon* spp., *Sarcocystis* spp. and/or *Emmonsia crescens*. Furthermore, the PUUV RNA-negative and anti-PUUV antibody-positive BF bank vole as well as the negative control vole had no pathological histological findings.

**Table 6:** Histologic findings in bank voles from BF.

	RT-PCR Pos ELISA Pos (Persistent Infection)
**Total**	12
**Lung** (a) Desquamated alveolar macrophages (b) Desquamated alveolar macrophages, BALT-hyperplasia (c) Few interstitial lymphocytes, macrophages and neutrophils	
	8/12 (67%)
	1/12 (8%)
	1/12 (8%)
**Glandula mandibularis** (a) Few interstitial lymphocytes (b) Few interstitial lymphocytes and plasma cells	
	1/10 (10%)
	1/10 (10%)
**Heart** (a) Few interstitial lymphocytes (b) Few interstitial lymphocytes, macrophages and plasma cells	
	1/12 (8%)
	1/12 (8%)

One of three and one of two RNA-negative and antibody-positive yellow-necked mice had interstitial mononuclear cells in the kidney and in the adrenal gland, respectively (Table 7). One RNA- and antibody-negative yellow-necked mouse was infected with *Emmonsia crescens* in the lung. Further pulmonal findings were desquamated alveolar macrophages and BALT hyperplasia. This animal also showed interstitial mononuclear infiltrates in the adrenal gland, salivary gland and heart (Table 7). Interstitial mononuclear infiltrates were also detected in the lung (1/2) or kidney (1/2) of RNA-negative and antibody-positive wood mice (Table 7). None of the yellow-necked mice and wood mice were infected with *Hepatozoon* spp. or *Sarcocystis* spp.

**Table 7:** Histologic findings in yellow-necked mice and wood mice.

	Yellow-Necked Mice		Wood Mice
	RT-PCR Neg ELISA Pos	RT-PCR Neg ELISA Neg	RT-PCR Neg ELISA Pos
**Total**	3 2 M, 1 F	1 1 M	2 1 M, 1 F
**Lung** (a) Desquamated alveolar macrophages, BALT-hyperplasia, *Emmonsia crescens* (b) Few interstitial mononuclear cells			
	0/3 (0%)	1/1 (100%)	0/2 (0%)
	0/3 (0%)	0/1 (0%)	1/2 (50%)
**Kidney** (a) Mild chronic non-suppurative interstitial nephritis (b) Few mononuclear cells in interstitium and renal pelvis (c) Few mononuclear cells in interstitium and renal pelvis, moderate glomerulosclerosis, interstitial fibrosis, multifocal mineralization, intratubular protein casts			
	1/3 (33%)	0/1 (0%)	0/2 (0%)
	1/3 (33%)	0/1 (0%)	1/2 (50%)
	1/3 (33%)	0/1 (0%)	0/2 (0%)
**Adrenal gland** (a) Few interstitial lymphocytes (b) Few interstitial lymphocytes, macrophages and plasma cells			
	1/2 (50%)	0/1 (0%)	0/1 (0%)
	0/2 (0%)	1/1 (100%)	0/1 (0%)
**Glandula mandibularis** (a) Few interstitial lymphocytes, macrophages and plasma cells			
	0/1 (0%)	1/1 (100%)	n.i.
**Heart** (a) Few interstitial lymphocytes and plasma cells			
	0/3 (0%)	1/1 (100%)	0/2 (0%)

n.i., not investigated; M, males; F, females.

## 4. Discussion

Bank voles serve as the natural host and reservoir for PUUV in Central, Western and Northern Europe [3]. Infected bank voles shed the virus via saliva, urine and feces [6] so that infection of humans occurs usually by inhalation of virus-contaminated dust or by biting [7]. To date, few data are available on the organ and cell tropism of PUUV in infected bank voles that could be analyzed by the detection of N protein or positive-strand viral RNA. This is the first study describing a broad organotropism for PUUV of the Central European and Russian clade in two different groups of naturally infected bank voles from the Western and Carpathian evolutionary lineage, respectively. This study in OS continued our monitoring study in this long-known PUUV endemic area [25]. The majority of PUUV RNA-positive animals showed also anti-PUUV antibodies, confirming a persistent infection. There are contrasting results regarding the difference in prevalence between the sexes. Some previous studies observed adult males to be the most frequently infected with PUUV [12,53,54,55,56]. However, Reil et al. 2017 [57] found no sex difference of PUUV prevalence in bank voles. An additional group of individuals showed only PUUV-reactive antibodies, but no viral RNA. This might be explained by the presence of maternal antibodies in juvenile animals or by a clearance of the infection in adult animals. Transmission of maternal antibodies from the dam to the newborn voles has been shown to result in an earlier maturation [58]. However, we cannot exclude an oscillation of the level of PUUV RNA during the course of infection being the reason for the lack of RNA detection, as previously reported [59]. In contrast to shedding patterns via excreta (high virus loads in urine and feces), the viral load in blood/saliva declined significantly after 7 months post infection. The BF bank voles, all females, were almost all found to be persistently infected; only a single individual was found to be affected by an acute infection.

The wild-trapped OS voles and the BF voles did not only differ in their evolutionary lineage origin (Western versus Carpathian) and the PUUV clade (Central European versus Russian), but also in the sex composition (only female animals in the BF group, but an equal male/female ratio in the OS group). This could also indicate differences in nutritional and immune status and genetic make-up, thus also influencing the susceptibility for PUUV infection. Interestingly, the tissue and cellular distribution of the PUUV N protein in principle was almost the same in both vole groups from OS and BF, with a few exceptions (see below). In summary, the N protein was found in the lung, kidney, small intestine, salivary gland, pancreas, liver, heart and cerebrum as described previously [60,61,62]. In addition, the N protein was detected for the first time in the stomach, testis, tongue and cerebellum. In BF voles, positive-strand viral RNA was found in the same organs as the N protein as well as in the caecum, colon ascendens, colon descendens, adrenal gland and brown adipose tissue, where the N protein was not detected. In some organs of OS and BF voles, discrepancies in the detection/absence of the N protein and respective positive-strand RNA were noted. This might be due to the differences in the animal groups per se as mentioned above but possibly also to the timepoint of infection, different susceptibility or ability of the respective cell type for virus replication, transcription and translation. The detection of viral N protein alone could also indicate protein uptake only, e.g., in macrophages. The detection of positive-strand viral RNA alone could also indicate complementary RNA at the respective localization that serves as a template for the synthesis of genomic negative-strand RNA, and not mRNA, which is necessary for the synthesis of the N protein.

Besides the broad organ tropism, a preference of the virus for the kidney and stomach was noted by IHC and ISH in most voles from both groups, even if only a few cells were infected in any organ that was positive for N protein or viral positive-strand RNA. Virus tropism for the kidney was expected, since hantaviruses can be excreted in the urine [7]. Furthermore, the N protein was found in the cerebrum of one third of the viral RNA and antibody-positive OS bank voles. Interestingly, despite the detection of viral RNA, the N protein was rarely detected in the lungs of wild or laboratory bank voles. Positive-strand viral RNA was detected in the lungs of all RNA and antibody-positive BF bank voles, respectively. This is remarkable since the lung is considered to be the primary replication site for hantaviruses [34]. In a previous study, the viral antigen had been detected in experimentally PUUV-infected bank voles from day 14 to day 270 post-intramuscular inoculation [61]. Whether the rare detection of N protein in this animal cohort in the lung might indicate a protective or virus-neutralizing function of anti-PUUV antibodies, represent a different stage of infection or be due to specific host or virus factors needs to be further investigated. The N protein was exclusively verified in the glandula mandibularis, tongue, liver and heart of voles from OS, whereas the glandula parotidea exclusively tested positive in animals of BF. These differences may be due to the already-mentioned differences in the two animal cohorts, but also the dose or timepoint of infection that we do not know due to the natural route of infection.

With regard to cellular tropism, it was striking that the viral N protein and positive-strand RNA were frequently found in cells that are classically incapable of secretion. These were endothelial cells, hepatic Kupffer cells, macrophages in the lung as well as neurons and glia cells. Similar observations were also made in previous studies. In contrast to our study, the N protein was additionally found in the spleen and renal tubular epithelial cells [60,61,62], both of which were negative for viral protein or RNA in both vole groups. In this study, viral positive-strand RNA and N protein were additionally found in various locations in the brain (plexus choroideus, stratum granulosum, moleculare and ganglionare), cardiomycoytes as well as in endocrine cells of the pancreas and adrenal gland for the first time. Moreover, in the OS and BF voles, positive-strand RNA and N protein occurred in fibrocytes, myocytes, myoepithelial cells, Ito cells or macrophages as well as in the podocytes or mesangial cells in the kidney. Whether this cell tropism indicates a specific strategy to circumvent the antiviral immune response or results from the broad organ and potential receptor tropism remains to be determined.

The identification of potential hantavirus entry receptors or attachment proteins was mainly based on investigations in human, monkey and laboratory animal-derived cell lines (for a review, see [63]). Initial in vitro studies resulted in the detection of several molecules representing candidate receptors or attachment proteins, i.e., certain integrins, complement accelerating factor (DAF/CD55) and gC1qR for different hantaviruses [64,65,66,67]. More recently, protocadherin-1 (PCDH1) was demonstrated as a virus clade-specific cellular attachment and entry factor by in vitro and in vivo studies in a Syrian hamster model and a CRISPR/Cas9-mediated knockout approach in a human endothelial cell line [68]. However, the authors raised the question about the attachment and entry receptors for PUUV and other Old World hantaviruses which still need to be proven in the natural PUUV bank vole reservoir [69].

In general, it is yet unknown how hantaviruses maintain a persistent infection in their reservoirs, despite the presence of neutralizing antibodies [70]. It might be possible that the infection of cells of the above-described non-secretory- and non-excretory organs contribute to virus persistence. The detection of hantavirus antigen in the nervous system of rats and in the endothelial cells of deer mice (*Peromyscus maniculatus*) has already been considered as an evasion of host immune defenses [71,72]. An analysis of saliva, urine and feces of naturally infected bank voles revealed that virus shedding through one route was intermittent, while the virus was shed through another route [59]. Whether viral presence in myocytes has an impact on the contractibility of the respective secretory organ remains speculative.

The reasons for this variable virus shedding pattern are not yet known [34,70], but could contribute to maintaining the virus in the reservoir population and transmission via various routes. However, this does not lead to clinical or pathomorphological findings in bank voles in the present and previous studies [60,61]. Individual factors, the respective PUUV strain, host genetic factors, age as well as population density and environmental factors can also have an influence on the level and pattern of virus shedding [34,59,73]. Thus, the reason for variable virus shedding might be a periodic reactivation of PUUV in non-secretory- and non-excretory cells with the subsequent relocation of replication in cells capable of virus shedding.

Regarding virus transmission within the reservoir population, the detection of N protein or positive-strand viral RNA in pulmonary epithelial cells most likely indicates airborne virus uptake. The presence of viral proteins in the acini of salivary glands, gastric epithelial cells and interstitial cells of the tongue could be due to aerogenic and/or oral PUUV uptake. It has been shown experimentally that Syrian golden hamsters (*Mesocricetus auratus*) can be infected with PUUV via the gastrointestinal tract, and it was suggested that this might also be possible under natural conditions [74]. Whether the oral route may have importance in bank voles needs to be further investigated. Regarding virus shedding, the detection of N protein or positive-strand RNA in the epithelial cells of the lung, salivary glands and stomach as well as in the acini of the pancreas and in the kidney may contribute to PUUV excretion via saliva, sputum, feces and urine. These data fit well to the known transmission routes [6] and already-described organ-specific cell types affected [60,61,62], whereas detection in the testis has not yet been described and needs further attention.

To date, no abnormal macroscopic and histological findings have been observed in naturally and experimentally PUUV-infected bank voles [60,61], which is in line with this study. Whether the detection of mononuclear cells in the lung can be associated with the presence of PUUV RNA or antigen remains elusive since they were found in the bank voles from both cohorts (OS and BF), and in the yellow-necked mice and wood mice in which no viral RNA was present. Thus, the presence of mononuclear infiltrates with concomitant negative molecular, serological and immunohistological results suggests other causes. Moreover, the detection of the N protein did not correlate with the inflammatory cell infiltrates in OS and BF voles. Thus, other causes such as, e.g., *Leptospira* spp., *Encephalitozoon cuniculi* and *Klossiella muris*, should be considered as potential etiological differential diagnoses [75,76,77].

In humans, there is an overlap of the infected organs and cell types to the above-described findings in the bank voles. For example, the kidneys are also considered to be the primary site of PUUV replication in humans [14]. As in bank voles, there is also a tropism of PUUV for glomerular cells (endothelial cells and podocytes) in humans [78]. Furthermore, there is a tropism for the tubular epithelial cells of infected patients. In humans, the infection of glomerular and tubular cells disturbs the structure and integrity of cell-to-cell contacts leading to proteinuria that is characteristic for PUUV-induced renal failure [78]. Comparably, in humans, a tropism for endothelial cells and macrophage-like cells or monocytes in the lung has been described [79]. In PUUV-infected humans, histologic lesions are relatively mild and unspecific, and an acute tubulointerstitial nephritis is the most common finding [80]. Only in severe human hantavirus disease cases interstitial mononuclear infiltrates have occurred in the lung [79]. It is believed that the infection of human endothelial cells leads to the accumulation of mononuclear immune cells, redistribution of tight junction proteins and necrosis of tubular epithelial cells [14,78]. As expected for the natural reservoir, no tubular necrosis was found in the bank voles examined.

Of note, all wild-trapped PUUV-infected OS bank voles, either acutely or persistently infected, exhibited a coinfection with *Hepatozoon* spp., *Sarcocystis* spp. or both, whereas all BF voles as well as anti-PUUV antibody-positive yellow-necked mice and wood mice were unaffected. Schizonts of *Hepatozoon* spp. in the lung [81] as well as *Sarcocystis* spp. in the brain [82] of bank voles have already been described similarly to *Emmonsia crescens* in the lung [83,84]. The latter was accompanied by granulomatous inflammation [85] as it was occasionally seen in this study. As mainly described in this study, Laakkonen et al. (2001) found only minimal tissue reactions in the lungs of bank voles caused by *Hepatozoon* spp. [81]. Desquamated alveolar macrophages and interstitial leukocyte infiltrates could be present in our study, whereas interstitial pneumonia was rarely detected in the wild-trapped OS voles. Nevertheless, it is conceivable that PUUV infection caused these lesions and BALT hyperplasia since all of these findings were also found in the *Hepatozoon* spp.-free BF bank voles. Whether the observed syncytia are caused by infection with *Hepatozoon* spp. or PUUV remains questionable, but low pH (pH 5.3) promotes the syncytia formation of some hantaviruses in vitro [86,87,88,89]. A very high prevalence of *Hepatozoon* spp. was already shown in bank voles but not in yellow-necked mice and wood mice, similar to this study [81,90,91,92]. Thus, bank voles seem to play a unique role as hosts for *Hepatozoon* species in Europe [92]. This was also found for *Sarcocystis* spp., but it remains unknown whether the low prevalence in other species is due to natural resistance, less contact with the parasite or due to death after infection [82]. It has already been speculated that coinfections with other agents, e.g., nematodes can increase the probability of a PUUV infection and shedding by modulating the immune response [34,35,93]. Whether this assumption could be adopted to the high protozoa prevalence in the wild-trapped PUUV-infected bank voles has to be further investigated, e.g., by analyses of wild bank vole cohorts from other locations. Alternatively, persistent PUUV infections might also enhance the susceptibility of voles for coinfections. The absence of these endoparasites in BF bank voles might be related to the origin of the bank voles or the breeding conditions and their consequences on host genetics.

Even if no PUUV RNA was detected in yellow-necked mice and wood mice, the detection of PUUV-reactive antibodies could indicate virus contact or spillover infection as already described in previous studies [2,4,94]. However, further evidence of PUUV infection, such as the detection of viral RNA or viral antigen, is lacking.

In summary, a broad organ and tissue tropism with a preference for the kidney and stomach was detected independently of the bank vole–PUUV cohorts. For the first time, the presence of a viral antigen and/or RNA was reported in the cerebellum, tongue, stomach, caecum, colon, testis, brown adipose tissue, endocrine pancreas and adrenal gland. The N protein and positive-strand RNA were found mainly in cells without secretory or excretory capacities, which may contribute to the maintenance of viral persistence. The detection of viral products in tissues capable of secretion or excretion, e.g., the glands, pancreas or testis, is relevant for virus shedding, whereas the detection in the airways and stomach could indicate airborne or oral uptake. Apart from the differences between the bank vole cohorts from OS and BF, representing two different virus–host systems, the tissue distribution and simultaneous presence of the N protein and positive-strand RNA were quite comparable. As expected, PUUV-associated pathological changes in naturally infected bank voles were absent in both cohorts, and were most likely due to other causes, except in the lungs. The differences between PUUV coinfections with *Hepatozoon* spp. and *Sarcocystis* spp. in both cohorts need to be demonstrated in future studies for additional bank vole host–PUUV systems. The results are a prerequisite for a deeper understanding of virus–host interactions in natural hantavirus reservoirs.

## Figures and Tables

**Table 1:** Detection of Puumala orthohantavirus (PUUV) RNA by RT-PCR, anti-PUUV IgG antibodies by ELISA and immunohistochemistry (IHC)-based detection of nucleocapsid (N)-protein in bank voles from the district Osnabrück (OS) and the breeding facility (BF).

	**No. of Positive/Total Number of Investigated Voles**		
	**RT-PCR Pos** **ELISA Pos** **(Persistent Infection)**		**RT-PCR Pos** **ELISA Neg** **(Acute Infection)**
	**OS**	**BF**	**OS**
	36/189	12/13	3/189
	**Detection of N-Protein by IHC**		
Total	19/29 (66%) 10/19 M, 9/19 F	9/12 (75%) 9/9 F	3/3 (100%) 2/3 M, 1/3 F
Cerebrum	7/21 (33%)	2/12 (17%)	0/2 (0%)
(a) Neuron	2/7 (29%)	2/2 (100%)	n.a.
(b) Glia cells	4/7 (57%)	2/2 (100%)	n.a.
(c) Endothelial cells	3/7 (43%)	0/2 (0%)	n.a.
Cerebellum	1/19 (5%)	2/12 (17%)	0/2 (0%)
(a) Stratum moleculare	0/1 (0%)	2/2 (100%)	n.a.
(b) Stratum ganglionare	0/1 (0%)	2/2 (100%)	n.a.
(c) Stratum granulosum	1/1 (100%)	2/2 (100%)	n.a.
Lung	1/28 (4%)	2/12 (17%)	1/3 (33%)
(a) Bronchiolar epithelial cells	1/1 (100%)	2/2 (100%)	0/1 (0%)
(b) Pneumocytes type I	0/1 (0%)	0/2 (0%)	1/1 (100%)
(c) IC with spindle-shaped and round-oval nuclei	1/1 (100%)	2/2 (100%)	1/1 (100%)
Glandula mandibularis (acini, IC with spindle-shaped nuclei)	1/16 (6%)	0/10 (0%)	0/1 (0%)
Glandula parotidea (acini)	0/16 (0%)	2/9 (22%)	0/1 (0%)
Tongue (IC with lancet to spindle-shaped nuclei)	2/14 (14%)	0/12 (0%)	1/1 (100%)
Liver	3/26 (12%)	0/12 (0%)	2/3 (67%)
(a) Kupffer cells	3/3 (100%)	n.a.	2/2 (100%)
(b) Cells with spindle-shaped nuclei at sinusoid periphery	0/3 (0%)	n.a.	1/2 (50%)
Pancreas	1/12	3/8	0/2 (0%)
(a) Acini	0/1 (0%)	3/3 (100%)	n.a.
(b) Islet cells of Langerhans	1/1 (100%)	1/3 (33%)	n.a.
(c) IC with spindle-shaped and round-oval nuclei	1/1 (100%)	2/3 (67%)	n.a.
Stomach/Pars non-glandularis (epithelial cells)	1/17 (6%)	2/12 (17%)	0/1 (0%)
Stomach/Pars glandularis (IC with lancet to spindle-shaped nuclei)	5/13 (38%)	8/11 (73%)	n.i.
Kidney (glomerulum cells *)	11/28 (39%)	4/12 (33%)	3/3 (100%)
Testis (sperm precursor cell)	1/8 (13%)	n.a.	0/1 (0%)
Heart (IC with lancet-shaped nuclei)	1/28 (4%)	0/11 (0%)	0/3 (0%)

No PUUV N protein was detected in the negative control bank vole. IC, interstitial cells; n.a., not applicable; n.i., not investigated; M, males; F, females; *, glomerulum cells include endothelial cells, mesangial cells and podocytes.

## Data Availability

All data are included within the manuscript and its supplementary file.

## Supplementary Materials

The following supporting information can be downloaded at: https://www.mdpi.com/article/10.3390/v15030612/s1, Figure S1: Design and workflow of this study. Figure S2: Phylogenetic consensus tree derived from partial cytochrome *b* gene sequences with 782 nucleotides in length. Figure S3: Phylogenetic consensus tree for Puumala orthohantavirus (PUUV) S segment sequences with a length of 465 nucleotides. Figure S4: No detection of PUUV nucleocapsid protein in tissues of the negative control bank vole. Figure S5: Detection of PUUV nucleocapsid protein in different tissues of BF (A,B) and OS (C) bank voles by IHC. Figure S6: No detection of PUUV positive-strand RNA in tissues of the negative control bank vole by RNAscope. Figure S7: No detection of PUUV nucleocapsid protein in the kidneys (A,B) of two male OS bank voles (RNA-negative, antibody-positive) infected with *Hepatozoon* spp. Table S1: Frozen and formalin-fixed samples of BF voles and wild rodents trapped in district Osnabrück during 2015–2017.
