# Supplementary material for: Tropism of Puumala orthohantavirus and Endoparasite Coinfection in the Bank Vole Reservoir
Source: Viruses. 2023 Feb 23;15(3):612. doi: 10.3390/v15030612 (PMC10058470; doi:10.3390/v15030612)
Supplement: Supplementary file 1 [file viruses-15-00612-s001.zip › viruses-2070072-supplementary.pdf]

Figure S1: Design and workflow of this study

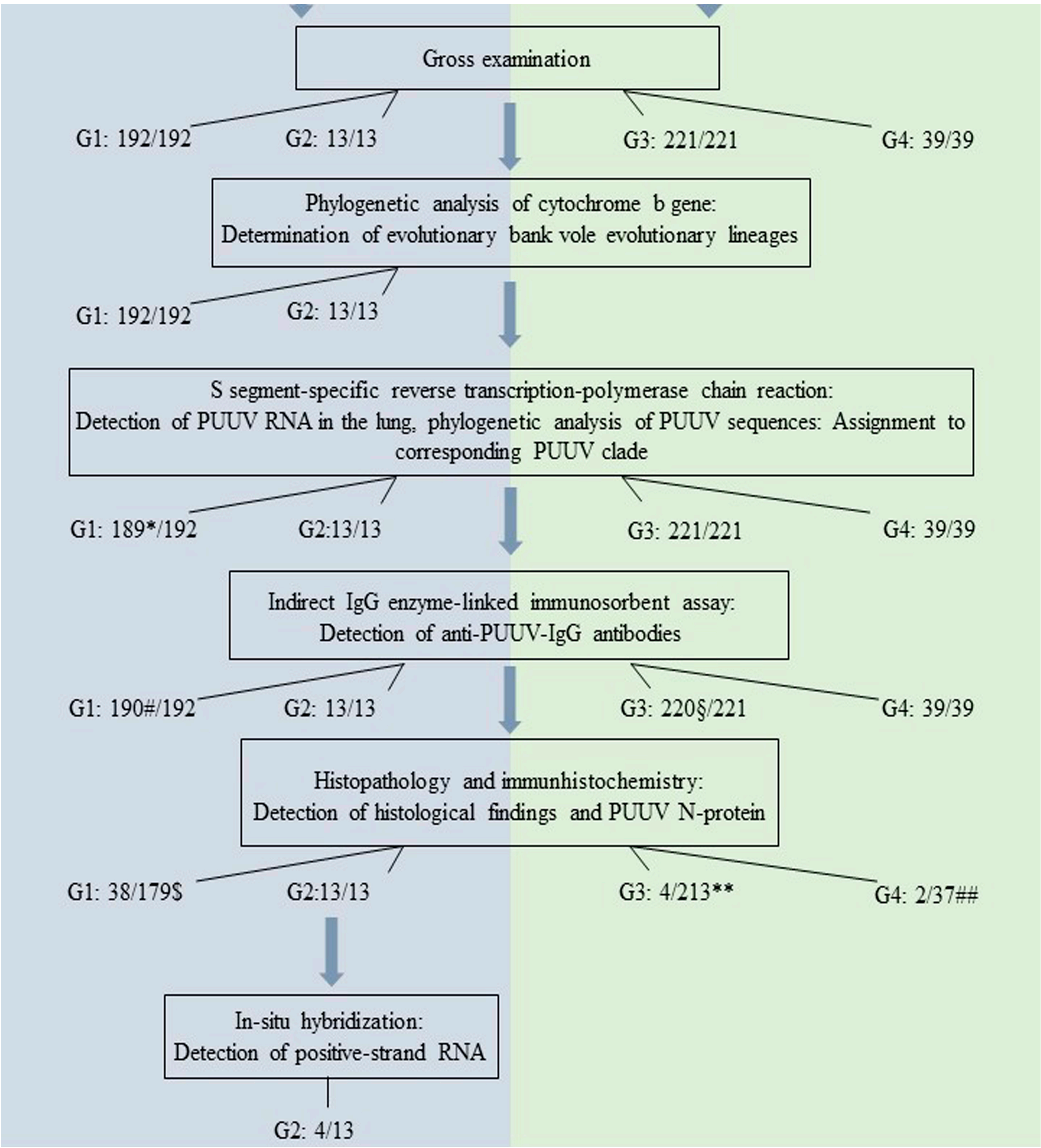

In three bank voles (*), the lungs were not available for examination. In two bank voles (#) and one yellow-necked mouse (§), no thoracic fluid could be obtained for the indirect ELISA. No formalin samples were taken from 13 bank voles ($), 8 yellow-necked mice (**) and 2 wood mice (##); G1, bank voles from OS; G2, BF voles; G3, yellow-necked mice from OS; G4, wood mice from OS; N, nucleocapsid; IgG, immunoglobulin G.

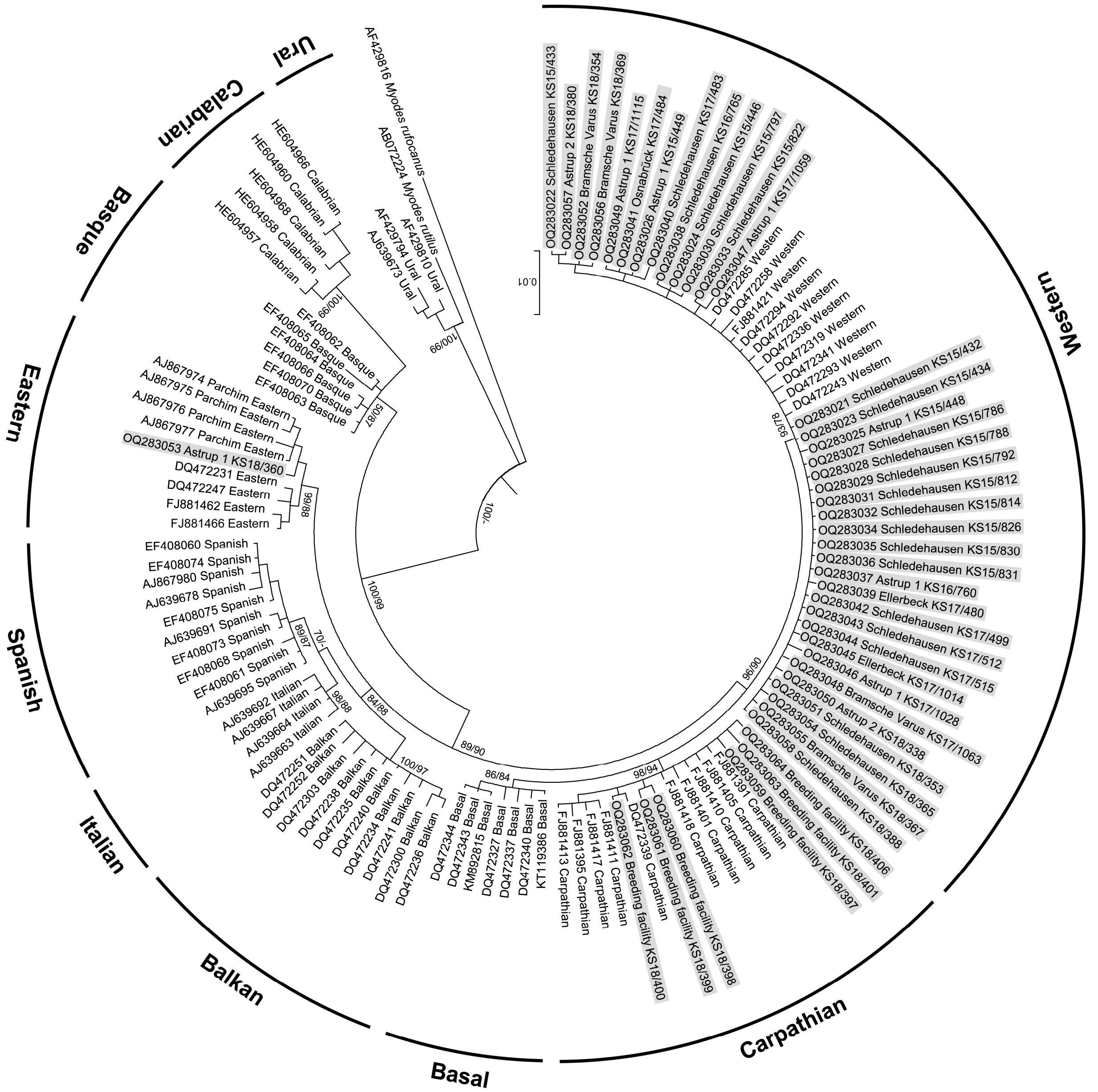

Figure S2: Phylogenetic consensus tree derived from partial cytochrome *b* gene sequences with 782 nucleotides in length. The Bayesian calculations were done with 8,000,000 generations and the TPM1uf substitution model with gamma distribution and invariant sites. The maximum likelihood tree was constructed with 1000 bootstrap replicates and the Jukes Cantor + CAT Model. Bootstrap values were only transferred to the Bayesian tree if branches were identical. Posterior probability values are given before and bootstrap values behind the slash. Novel sequences are indicated in grey.

Treatment of identical sequences according to the following pattern. The first mentioned identical sequence was retained, whereas the latter were excluded from phylogenetic tree reconstruction. Region of Osnabrück (Germany): KS15/432 identical to KS15/435, KS15/436, KS15/437, KS15/438, KS15/439, KS15/440, KS15/441, KS15/442, KS15/443, KS15/444, KS15/445, KS15/447, KS15/451, KS15/453, KS15/454, KS15/774, KS15/775, KS15/776, KS15/778, KS15/779, KS15/780, KS15/781, KS15/782, KS15/783, KS15/784, KS15/785, KS15/787, KS15/789, KS15/790, KS15/791, KS15/793, KS15/794, KS15/795, KS15/809, KS15/811, KS15/813, KS15/815, KS15/816, KS15/817, KS15/819, KS15/820, KS15/821, KS15/823, KS15/824, KS15/829, KS16/759, KS16/764, KS16/766,

KS16/767, KS16/770, KS16/771, KS17/479, KS17/485, KS17/494, KS17/504, KS17/506, KS17/508, KS17/1032, KS17/1035, KS17/1046, KS17/1048, KS17/1052, KS17/1062, KS17/1064, KS17/1065, KS17/1070, KS17/1072, KS17/1074, KS17/1084, KS17/1093, KS17/1094, KS17/1095, KS17/1098, KS17/1117, KS17/1118, KS18/337, KS18/339, KS18/340, KS18/341, KS18/343, KS18/344, KS18/345, KS18/347, KS18/349, KS18/351, KS18/352, KS18/355, KS18/356, KS18/357, KS18/358, KS18/359, KS18/362, KS18/363, KS18/364, KS18/366, KS18/368, KS18/371, KS18/372, KS18/373, KS18/374, KS18/375, KS18/376, KS18/378, KS18/379, KS18/381, KS18/383, KS18/384, KS18/385, KS18/386, KS18/390, KS18/391, KS18/392, KS18/393, KS18/394 and KS18/396. KS15/433 identical to KS15/450, KS15/777, KS16/769, KS17/1089, KS17/1092, KS17/1119, KS18/342, KS18/348 and KS18/377. KS15/446 identical to KS17/500 and KS17/1044. KS15/448 identical to KS17/503, KS17/507, KS17/1066 and KS18/361. KS15/449 identical to KS15/452, KS16/756, KS17/491, KS17/493 and KS18/382. KS15/788 identical to KS15/827. KS15/792 identical to KS15/796, KS15/828, KS16/768 and KS17/489. KS17/1014 identical to KS17/1060. KS17/1057 identical to KS17/1116 and KS18/389. KS17/1059 identical to KS18/346. KS18/338 identical to KS18/370. KS18/360 identical to KS18/395. KS18/369 identical to KS18/387. KS15/822 is identical to KS15/825. KS17/483 identical to KS17/1057. KS17/499 identical to KS17/1045. Colony (Poland): KS18/398 identical to KS18/404. KS18/400 identical to KS18/402, KS18/403, KS18/404 and KS18/405.

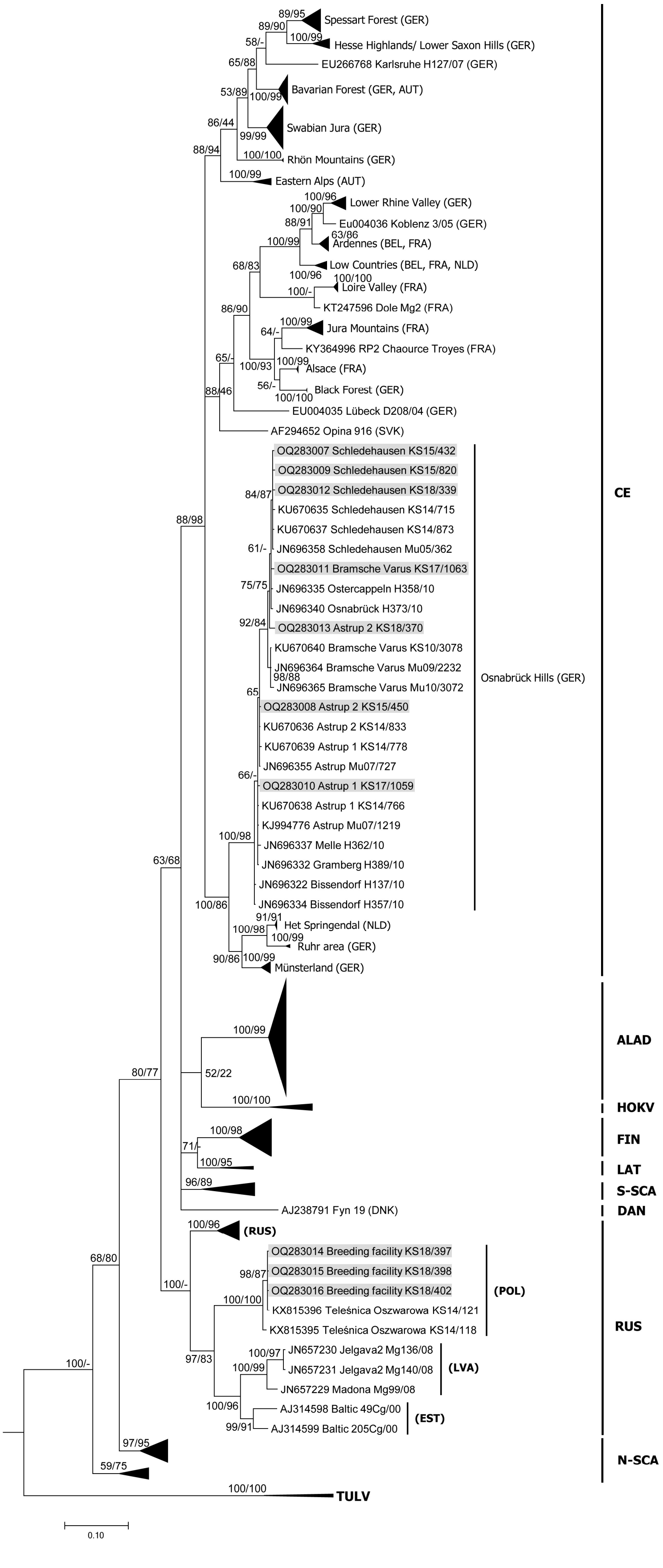

Spessart Forest (GER)
Hesse Highlands/ Lower Saxon Hills (GER)
EU266768 Karlsruhe H127/07 (GER)
Bavarian Forest (GER, AUT)
Swabian Jura (GER)
Rhön Mountains (GER)
Eastern Alps (AUT)
Lower Rhine Valley (GER)
Eu004036 Koblenz 3/05 (GER)
Ardennes (BEL, FRA)
Low Countries (BEL, FRA, NLD)
Loire Valley (FRA)
KT247596 Dole Mg2 (FRA)
Jura Mountains (FRA)
KY364996 RP2 Chaource Troyes (FRA)
Alsace (FRA)
Black Forest (GER)
EU004035 Lübeck D208/04 (GER)
AF294652 Opina 916 (SVK)
OQ283007 Schledehausen KS15/432
OQ283009 Schledehausen KS15/820
OQ283012 Schledehausen KS18/339
KU670635 Schledehausen KS14/715
KU670637 Schledehausen KS14/873
JN696358 Schledehausen Mu05/362
OQ283011 Bramsche Varus KS17/1063
JN696335 Ostercappeln H358/10
JN696340 Osnabrück H373/10
OQ283013 Astrup 2 KS18/370
KU670640 Bramsche Varus KS10/3078
JN696364 Bramsche Varus Mu09/2232
JN696365 Bramsche Varus Mu10/3072
OQ283008 Astrup 2 KS15/450
KU670636 Astrup 2 KS14/833
KU670639 Astrup 1 KS14/778
JN696355 Astrup Mu07/727
OQ283010 Astrup 1 KS17/1059
KU670638 Astrup 1 KS14/766
KJ994776 Astrup Mu07/1219
JN696337 Melle H362/10
JN696332 Gramberg H389/10
JN696322 Bissendorf H137/10
JN696334 Bissendorf H357/10
Osnabrück Hills (GER)
Het Springendal (NLD)
Ruhr area (GER)
Münsterland (GER)
CE
ALAD
HOKV
FIN
LAT
S-SCA
AJ238791 Fyn 19 (DNK)
DAN
(RUS)
OQ283014 Breeding facility KS18/397
OQ283015 Breeding facility KS18/398
OQ283016 Breeding facility KS18/402
KX815396 Teleśnica Oszwarowa KS14/121
KX815395 Teleśnica Oszwarowa KS14/118
(POL)
JN657230 Jelgava2 Mg136/08
JN657231 Jelgava2 Mg140/08
JN657229 Madona Mg99/08
(LVA)
AJ314598 Baltic 49Cg/00
AJ314599 Baltic 205Cg/00
(EST)
RUS
N-SCA
TULV
0.10

Figure S3: Phylogenetic consensus tree for Puumala orthohantavirus (PUUV) S segment sequences with a length of 465 nucleotides. The tree is based on Bayesian calculations with 10,000,000 generations and the TIM2 substitution model with gamma distribution and invariant sites. The maximum likelihood tree was established with 1000 bootstrap replicates using the Jukes Cantor + CAT Model. To construct a consensus tree, bootstrap values were only transferred to the Bayesian tree in case of branch consistency. Posterior probability values are given before and bootstrap values behind the slash. Novel sequences are highlighted by a grey background.
PUUV lineages: ALAD Alpe-Adrian, CE Central European, DAN Danish, FIN Finnish, LAT Latvian, N-SCA North-Scandinavian, RUS Russian, S-SCA South-Scandinavian.
Country abbreviations: (AUT) Austria, (BEL) Belgium, (DNK) Denmark, (EST) Estonia, (FRA) France, (GER) Germany, (LVA) Latvia, (NLD) the Netherlands, (POL) Poland, (RUS) Russia, (SVK) Slovakia.
Virus abbreviations: TULV Tula virus, HOKV Hokkaido virus.
Identical sequences were treated according to the following strategy. While the first mentioned identical sequence was preserved, the latter were excluded from phylogenetic tree reconstruction. Region of Osnabrück (GER): KS15/432 identical to KS15/434, KS15/436, KS15/438, KS15/443, KS15/444, KS15/447, KS15/774, KS15/777, KS15/796, KS15/812, KS15/817, KS15/824, KS15/825, KS15/827, KS16/766, KS16/767, KS16/771, KS17/1032, KS17/1072, KS17/1074, KS17/1084, KS17/1119, KS18/345, KS18/373, KS18/386, KS18/388 and KS18/396. KS15/450 identical to KS15/451 and KS15/453. KS17/1059 identical to KS17/1115. Colony (POL): KS18/397 identical to KS18/400 and KS18/401. KS18/398 identical to KS18/399. KS18/402 identical to KS18/403, KS18/404, KS18/405 and KS18/406.

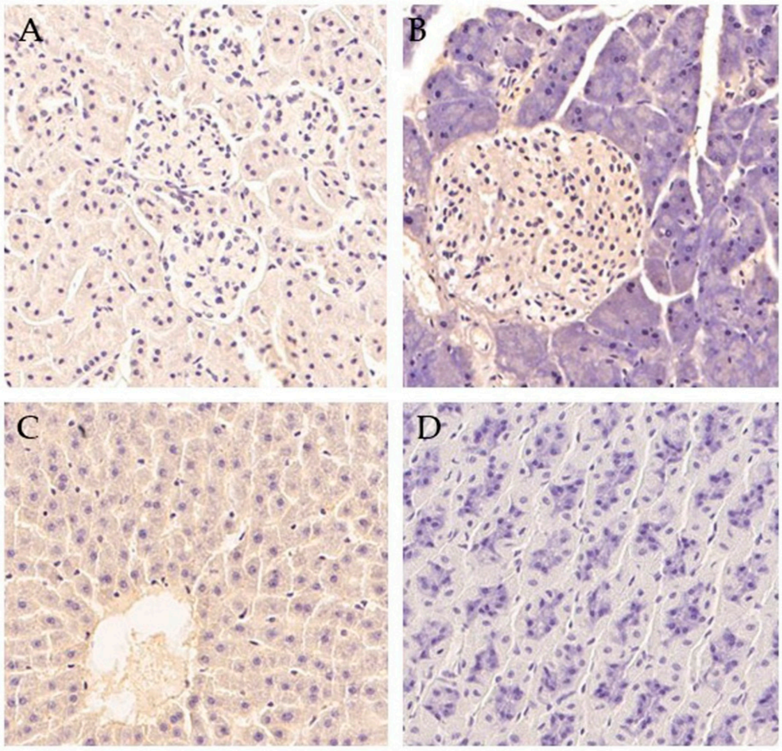

Figure S4: No detection of PUUV nucleocapsid protein in tissues of the negative control bank vole. (A) Kidney; (B) Pancreas; (C) Liver; (D) Pars glandularis of the stomach. Total magnification: A-D: 200x.

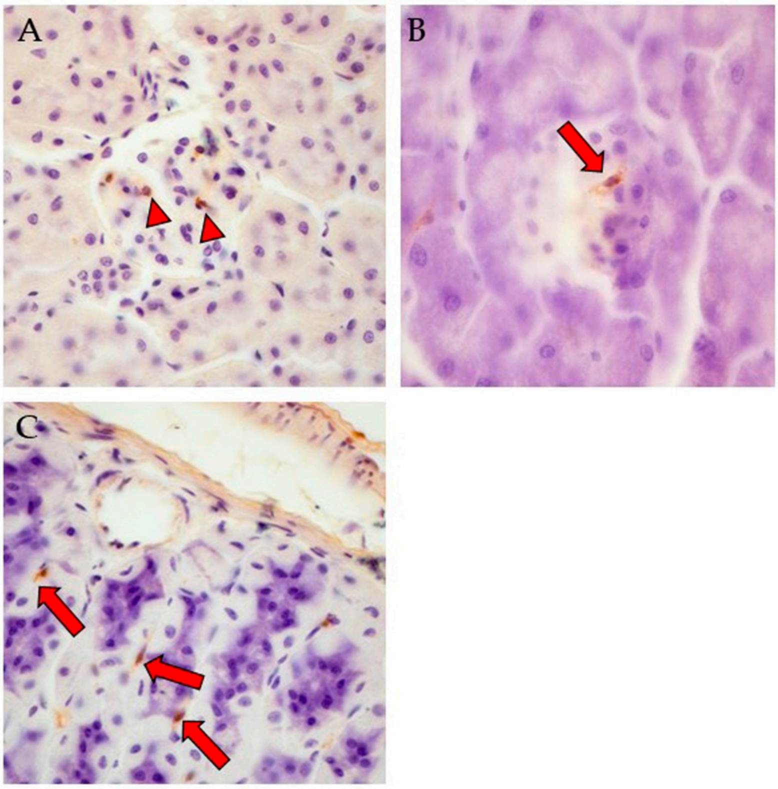

Figure S5: Detection of PUUV nucleocapsid protein in different tissues of BF (A, B) and OS (C) bank voles by IHC. A: female, RNA-positive, antibody-positive, kidney: positive glomerulum cells (red arrowheads); B: female, RNA-positive, antibody-positive, endocrine pancreas: positive islet cell of Langerhans (red arrow); C: male, RNA-positive, antibody-positive, pars glandularis of the stomach: viral antigen in interstitial cells with lancet to spindle-shaped nuclei (red arrows). Total magnification: A-C: 400x.

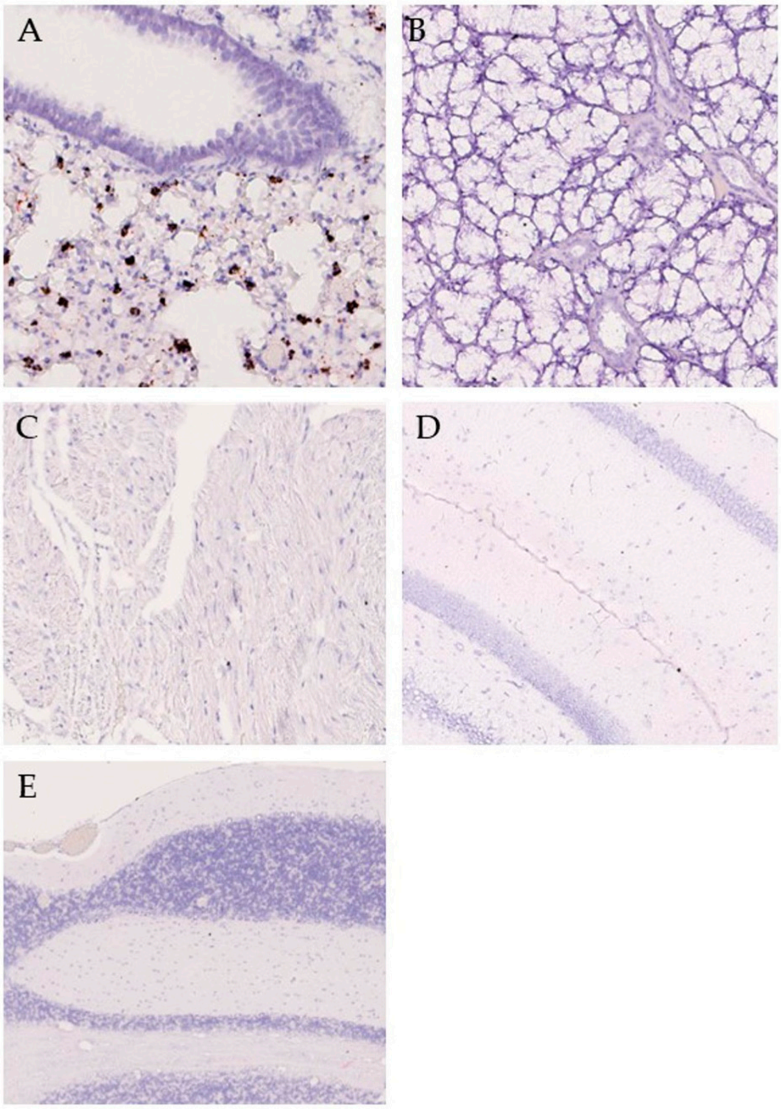

Figure S6: No detection of PUUV positive-strand RNA in tissues of the negative control bank vole by RNAscope. (A) Lung; (B) Glandula submandibularis; (C) Heart; (D) Cerebrum; (E) Cerebellum. Total magnification: A: 200x, B-E: 100x.

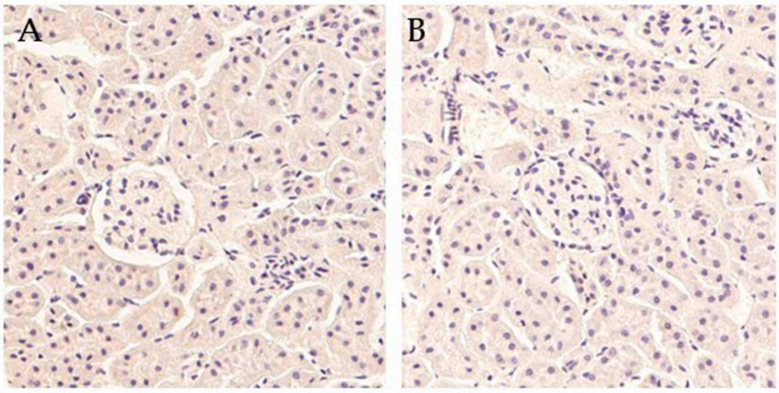

Figure S7: No detection of PUUV nucleocapsid protein in the kidneys (A, B) of two male OS bank voles (RNA-negative, antibody-positive) infected with *Hepatozoon* spp. Total magnification: A, B: 200x.

Table S1: Frozen and formalin-fixed samples of BF voles and wild rodents trapped in district Osnabrück during 2015-2017.

<table>
<tr><th colspan="3"></th><th>BF voles</th></tr>
<tr><th colspan="4">Bank voles, yellow-necked mice and wood mice from OS</th></tr>
<tr><th>Spring and Autumn 2015</th><th>Spring 2016</th><th>Autumn 2016 and Spring 2017</th><th>Autumn 2017</th></tr>
<tr><td colspan="4">Brain</td></tr>
<tr><td colspan="4">Heart</td></tr>
<tr><td colspan="4">Lung</td></tr>
<tr><td colspan="4">Liver</td></tr>
<tr><td colspan="4">Spleen</td></tr>
<tr><td colspan="4">Kidney</td></tr>
<tr><td colspan="4">Colon descendens</td></tr>
<tr><td></td><td colspan="3">Conchae with nasal mucosa</td></tr>
<tr><td></td><td colspan="3">Salivary glands</td></tr>
<tr><td></td><td colspan="3">Tongue</td></tr>
<tr><td></td><td colspan="3">Stomach</td></tr>
<tr><td></td><td colspan="3">Duodenum</td></tr>
<tr><td></td><td colspan="3">Jejunum</td></tr>
<tr><td></td><td colspan="3">Cecum</td></tr>
<tr><td></td><td colspan="3">Colon ascendens</td></tr>
<tr><td></td><td colspan="3">Brown adipose tissue</td></tr>
<tr><td></td><td colspan="3">White adipose tissue</td></tr>
<tr><td></td><td colspan="3">Urinary bladder</td></tr>
<tr><td></td><td colspan="3">Uterus</td></tr>
<tr><td></td><td colspan="3">Ovary</td></tr>
<tr><td></td><td colspan="3">Testis</td></tr>
<tr><td colspan="3"></td><td>Skin from the neck</td></tr>
<tr><td>Pancreas</td><td colspan="3">Pancreas</td></tr>
<tr><td colspan="2">Adrenal gland</td><td colspan="2">Adrenal gland</td></tr>
<tr><td></td><td>Esophagus</td><td colspan="2">Esophagus</td></tr>
<tr><td></td><td colspan="3">Lymphonodus mandibularis</td></tr>
<tr><td></td><td colspan="3">Skin from the head</td></tr>
<tr><td colspan="4">Thoracic fluid</td></tr>
<tr><td colspan="2"></td><td colspan="2">Trachea</td></tr>
</table>

Based on the results of initial organ spectrum evaluation, the scope of the frozen samples was constantly expanded. Green background: Served as a frozen and formalin-fixed sample; orange background: preserved as a formalin-fixed sample; blue background: Preserved as a frozen sample. In 2015, the brain (formalin-fixed and frozen sample) was not removed from 45 OS voles, 7 yellow-necked mice and one wood mouse, and the colon descendens (formalin sample) was not taken from 50 OS voles, 7 yellow-necked mice and one wood mouse. In the same year, no formalin samples were served from 13 OS voles, 8 yellow-necked mice and 2 wood mice.
